# Regioselective Electrophilic Aromatic Bromination: Theoretical Analysis and Experimental Verification

**DOI:** 10.3390/molecules19033401

**Published:** 2014-03-20

**Authors:** Hui-Jing Li, Yan-Chao Wu, Jian-Hong Dai, Yan Song, Runjiao Cheng, Yuanyuan Qiao

**Affiliations:** 1 School of Marine Science and Technology, Harbin Institute of Technology at Weihai, Weihai 264209, China; 2 School of Materials Science and Engineering, Harbin Institute of Technology at Weihai, Weihai 264209, China; 3 Central Laboratory, College of Chemistry, and Computational Center for Molecular Science, Nankai University, Tianjin 300071, China

**Keywords:** electrophilic aromatic bromination, selectivity, *ab initio*, natural products

## Abstract

Electrophilic aromatic bromination is the most common synthetic method used to prepare aryl bromides, which are very useful intermediates in organic synthesis. To understand the experimental results in electrophilic aromatic brominations, *ab initio* calculations are used here for a tentative analysis of the positional selectivity. The calculated results agree well with the corresponding experimental data, and the reliability of the resulting positional selectivity was verified by the corresponding experimental data.

## 1. Introduction

Aryl bromides have found widespread utility as substrates for Pd-, Ni-, and Cu-catalyzed cross-coupling reactions to form diverse C–C, C−N, C−O, and C−S bonds [1,2,3,4,5,6,7,8,9]. Moreover, aryl bromides have been used as classical precursors to organolithium and Grignard reagents as well as in benzyne generation and nucleophilic aromatic substitution [10,11]. The most common synthetic method for preparing aryl bromides is electrophilic aromatic bromination, which continues to be an area of focus in synthetic chemistry because aryl bromides are very useful intermediates in the production of drugs, pharmaceuticals, agrochemicals, pigments, photographic materials, and various functional natural products [1,2,3,4,5,6,7,8,9,10,11,12,13,14,15,16,17,18,19,20,21,22,23,24,25,26,27,28,29,30,31,32,33,34,35,36,37]. This makes the development of regioselective electrophilic aromatic brominations a high priority. Accordingly, many regioselective electrophilic aromatic brominations have been developed. Tetraalkylammonium tribromides are highly *para*-selective for bromination of phenols [12]. LDH-CO_3_^2−^-Br_3_^−^ can be used for *para*-selective monobromination of some aromatic compounds [13]. Zeolites induce high *para*-selectivity for electrophilic bromination of substrates akin to toluene [14,15,16,17,18,19,20,21,22]. *N*-bromosuccinimide (NBS)/silica gel is also a good brominating agent for regioselective electrophilic aromatic brominations [23,24]. Bromine (Br_2_), produced *in situ* in either a Cu(NO_3_)_2_/HBr/O_2_/H_2_O system [25] or LiBr/ceric ammonium nitrate (CAN) system [26], displays similar regioselectivity in electrophilic aromatic brominations. *N*-bromosuccinimide, in either ionic liquids [27] or THF [28], is highly regioselective for electrophilic aromatic brominations, whereas bromodimethylsulfonium bromide [29] and 1,3-dibromo-5,5-dimethyihydantoin [30] are less regioselective for electrophilic aromatic brominations in comparison with NBS. Although direct comparison is not always valid due to the different reaction conditions, the positional selectivity of electrophilic aromatic bromination is quite clear. For an analysis of the inherent positional selectivity of electrophilic aromatic brominations, theoretical calculations may be required as they might provide insightful quantitative information that would be difficult to obtain by experimental methods.

**Scheme 1:**
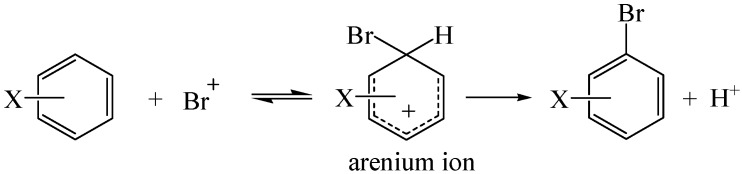
Electrophilic aromatic bromination.

Usually, an electrophilic aromatic bromination is considered to occur via a stepwise mechanism (Scheme 1) [31,32,33,34,35,36,37]. A π complex [38] or a radical ion pair [39] may or may not be directly involved in an electrophilic aromatic bromination mechanism and its rapidly reversible formation is usually not the rate-determining step [31,32,33,34,35,36,37]. However, for an electrophilic aromatic bromination to occur, a cationic reaction intermediate called an arenium ion, known as a σ complex or Wheland intermediate [40], is usually formed [31]. The formation of bromoarenium ion is difficult due to the loss of the inherent stability associated with aromaticity and is usually the rate-determining step, in which the transition state comes later on the reaction coordinate and is closer in energy to the arenium ion [41]. According to the Hammond postulate [42], the rate-determining transition state resemble the arenium ion, so that factors stabilizing bromoarenium ion also stabilize the transition state and lower the activation energy, and thereby usually favor the electrophilic aromatic bromination.

**Figure 1:**
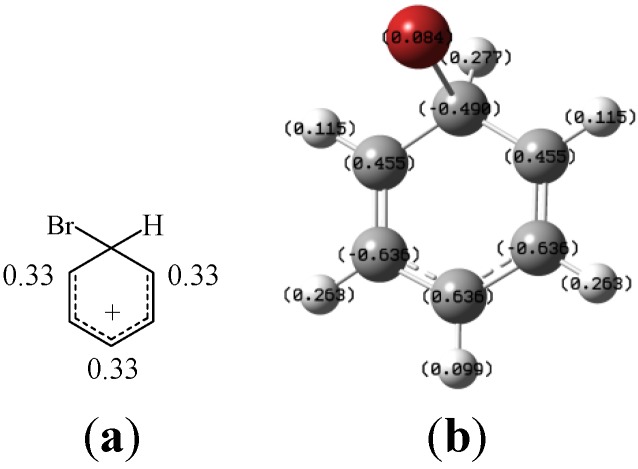
Charge distribution of the arenium ion based on (**a**) Hückel molecular orbital method and (**b**) the *ab initio* calculations via GAUSSIAN 09 program package. Values on C and H atoms in (b) indicate the atomic charge assignments (the positive or negative sign implies that the atom donates or accepts charges).

Substituents can influence the product distribution by favoring the formation of one arenium ion over another. Based on Hückel molecular orbital theory, the positive charge of the arenium ion is equally delocalized at the positions that are *ortho* and *para* to the site of the substitution (Figure 1) [31,32,33,34,35,36,37]. Therefore, a π-donor substituent at one of these positions stabilizes the arenium ion and also stabilizes the transition state and lowers the activation energy necessary for the electrophilic aromatic substitution, and thus acts as an *ortho*/*para* directing group. In contrast, a π-acceptor substituent at one of these positions destabilizes the arenium ion and increases the activation energy necessary to attain the transition state, and thus acts as a *meta* directing group. The resulting principles, known as the Holleman rules [43], are easily explained by comparison of the corresponding resonance structures [32].

**Scheme 2:**
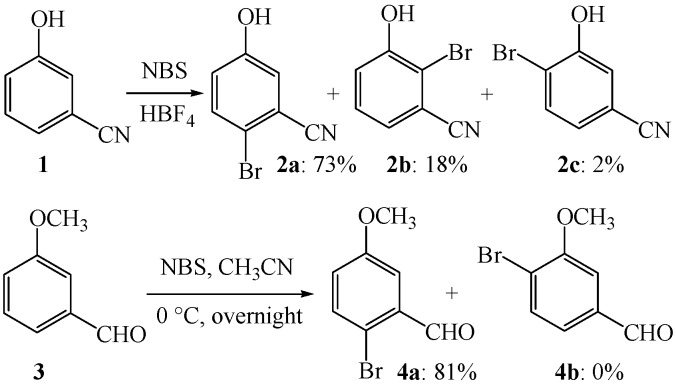
Electrophilic aromatic brominations of 3-hydroxybenzonitrile and 3-methoxy-benzaldehyde.

With only Holleman rules in hands, some related experimental results in electrophilic aromatic brominations cannot be explained well. For example, the electrophilic aromatic bromination of 3-hydroxybenzonitrile (**1**, Scheme 2) afforded 2-bromo-5-hydroxybenzonitrile (**2a**) and 2-bromo-3-hydroxybenzonitrile (**2b**) in 73% and 18% yields, respectively.

In contrast, 4-bromo-3-hydroxybenzonitrile (**2c**) was isolated in only 2% yield [44]. Similarly, the electrophilic aromatic bromination of 3-methoxybenzaldehyde (**3**) afforded exclusively 2-bromo-5-methoxybenzaldehyde (**4a**) in 81% yield, while the potential isomer of 4-bromo-3-methoxybenzaldehyde (**4b**) was not formed at all [45,46]. As witnessed in these two examples, a π-donor substituent (πDS) usually tends to facilitate its *para* electrophilic aromatic bromination, whereas a π-acceptor substituent (πAS) usually tends to prevent the corresponding *para* electrophilic aromatic bromination. According to Holleman rules, compounds **2c** and **4b** should also be the major products, yet they are not. Why? To understand these experimental results, *ab initio* calculations are used here for a tentative analysis of the positional selectivity. *Ab initio* studies of the bromination of benzene have already been carried out previously to discuss the reaction pathways [33]. Herein the results based on *ab initio* calculations are used to distinguish the *ortho*/*para* positional selectivity.

## 2. Results and Discussion

Calculating the charge distribution in the arenium ion by *ab initio* calculations via the GAUSSIAN 09 program package [47] did provide some insightful quantitative information. The atomic charge assignments on the C and H atoms of the arenium ion are shown in Figure 1b, where the positive or negative sign implies that the atom donates or accepts charges. The calculations indicated that the overall magnitude of the electron deficiency of the arenium ion over the various positions follows the order of *para* > *ortho* > *meta*. Without influence of any other factors, a π-donor substituent would direct an electrophilic aromatic bromination to the position in the preferential order of *para* > *ortho* > *meta*, whereas a π-acceptor substituent would direct an electrophilic aromatic bromination to the position in the preferential order of *meta* > *ortho* > *para*.

**Scheme 3:**
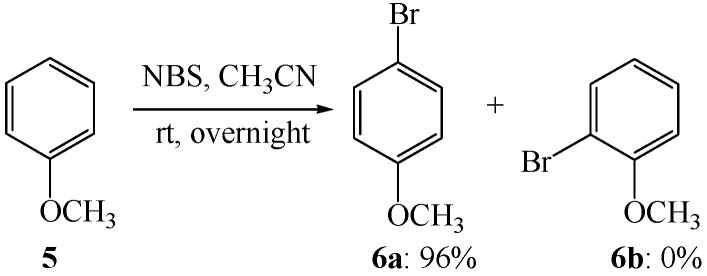
Electrophilic aromatic bromination of anisole.

The analysis is in good agreement with the experimental observations. For example, methoxyl group is a π-donor substituent due to a resonance effect which helps to delocalize the positive charge of the arenium ion onto the oxygen atom. Accordingly, the electrophilic aromatic bromination of anisole (**5**) under the reported conditions [45,46] afforded exclusively 1-bromo-4-methoxybenzene (**6a**) in 96% yield, and the potential product of 1-bromo-2-methoxybenzene (**6b**) was not detected (Scheme 3). As in the case of anisole, the electrophilic aromatic bromination of a wide range of arenes with NBS in acetonitrile proceeded readily and was found to be highly *para*-selective with respect to the most activating substituent, in instances where the *para* position is blocked, bromination occurred *ortho* to the most activating substituent [45,46]. Similar positional selectivity was usually found when the reactions were performed in the other electrophilic aromatic bromination systems [27,28].

**Figure 2:**
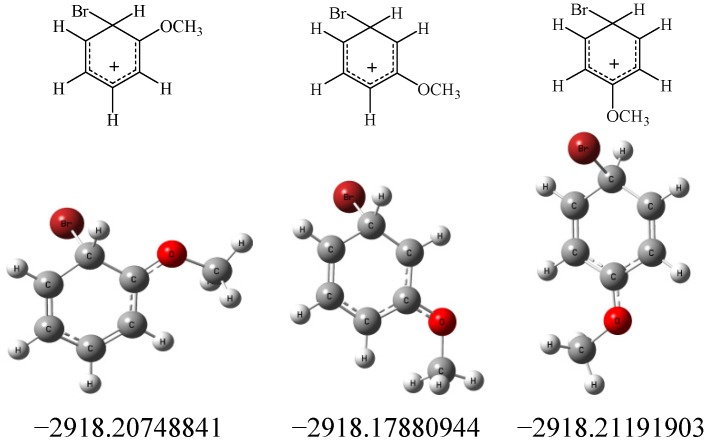
Total of electronic energies (Hartree) of the transition-states of the isomer products in the electrophilic aromatic bromination of anisole.

The reliability of the above *ab initio* calculations could be verified by the energy differences of transition-states associated with the corresponding isomer products. Take the electrophilic aromatic bromination of anisole as example, the energy of transition-states associated with the isomer products have been also performed based on *ab initio* calculations via GAUSSIAN 09 program package [47]. As shown in Figure 2, the energy of the arenium ion associated with the *para* isomer is lower than that associated with the *ortho* isomer, and the energy of the arenium ion associated with *meta* isomer is highest. The study supports the same positional selectivity trends deduced from the viewpoint of the charge distribution of the arenium ion.

**Figure 3:**
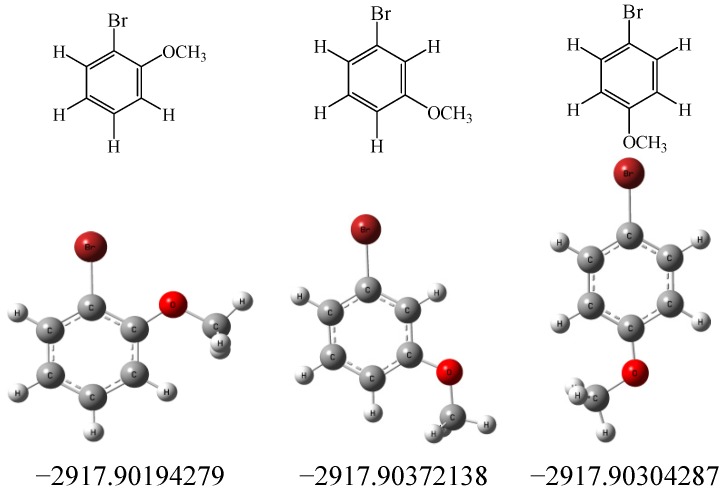
Total of electronic energies (Hartree) of the isomer products in the electrophilic aromatic bromination of anisole. Energies of isomer product + H^+^ (large distance between H and isomer product) are −2918.42094279 (*ortho* isomer), −2918.42272138 (*meta* isomer) and −2918.42204287 (*para* isomer), respectively.

To understand the high positional selectivity in the above electrophilic aromatic bromination of anisole, the energy differences of the isomer products have also been calculated based on *ab initio* calculations via GAUSSIAN 09 program package [47]. As shown in Figure 3, the energy of the *para* isomer is lower than that of the *ortho* isomer, indicating the *para* isomer is more stable than the *ortho* isomer, which also supports the high *para*/*ortho* selectivity mentioned above.

**Scheme 4:**
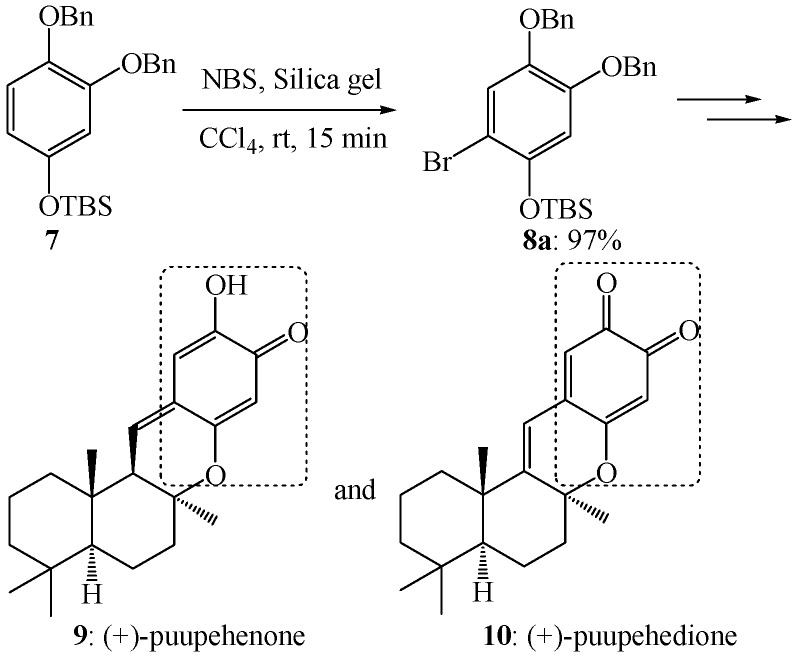
Electrophilic aromatic bromination of compound **7** and its experimental application.

As the results based on *ab initio* calculations agree well with the related experimental observations in electrophilic aromatic brominations, the resulting positional selectivity should be well considered in the synthesis of various aryl bromides, which are very useful intermediates in organic synthesis. For example, the positional selectivity has facilitated the synthesis of various bioactive natural products. As benzyloxyl and *tert*-butyldimethylsilyloxy are quite comparable activating substituents, the electrophilic aromatic bromination of compound **7** should give compound **8a** as the major isomer according to the positional selectivity mentioned above (Scheme 4). Indeed, treatment of compound **7** with *N*-bromosuccinimide and silica gel in carbon tetrachloride at room temperature for 15 min afforded exclusively the isomer **8a** in 97% yield [48,49]. This regiospecific electrophilic aromatic bromination facilitated the synthesis of (+)-puupehenone (**9**) and (+)-puupehedione (**10**, Scheme 4) [48,49].

**Scheme 5:**
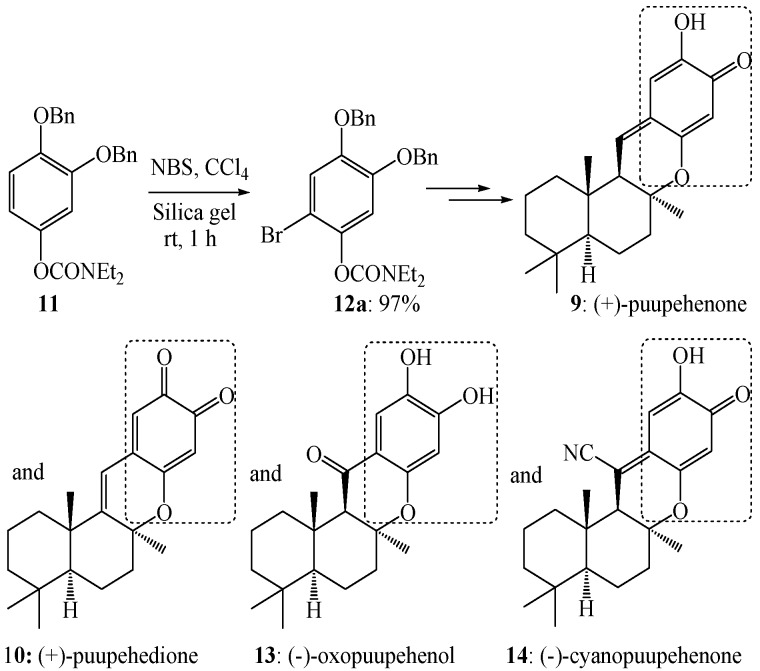
Electrophilic aromatic bromination of compound **11** and its experimental applications.

As in the case of compound **7**, the electrophilic aromatic bromination of compound **11** with *N*-bromosuccinimide and silica gel in carbon tetrachloride at room temperature for 1 h afforded exclusively compound **12a** in 97% yield, which in turn facilitated the synthesis of (+)-puupehenone (**9**), (+)-puupehedione (**10**), (−)-oxopuupehenol (**13**) and (−)-cyanopuupehedione (**14**, Scheme 5) [50].

**Scheme 6:**
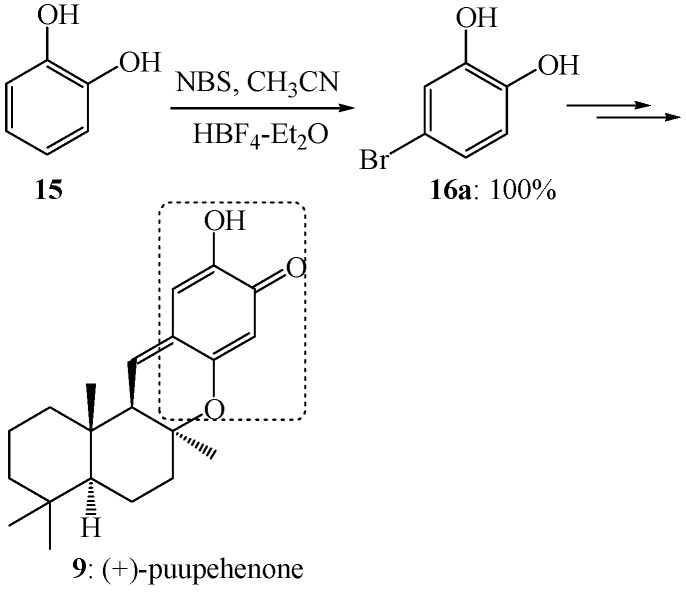
Electrophilic aromatic bromination of compound **15** and its experimental application.

As shown in Scheme 6, high potential selectivity in the electrophilic aromatic bromination of catechol (**15**) was accomplished by carefully controlling the reaction temperature. The reaction of catechol (**15**) with *N*-bromosuccinimide and fluoroboric acid in acetonitrile was performed at −30 °C, the reaction mixture was allowed to warm up to room temperature, and then stirred at this temperature overnight to afford 4-bromobenzene-1,2-diol (**16a**) in 100% yield. This regiospecific electrophilic aromatic bromination facilitated an elegant synthesis of (+)-puupehenone (**9**) [51].

**Scheme 7:**
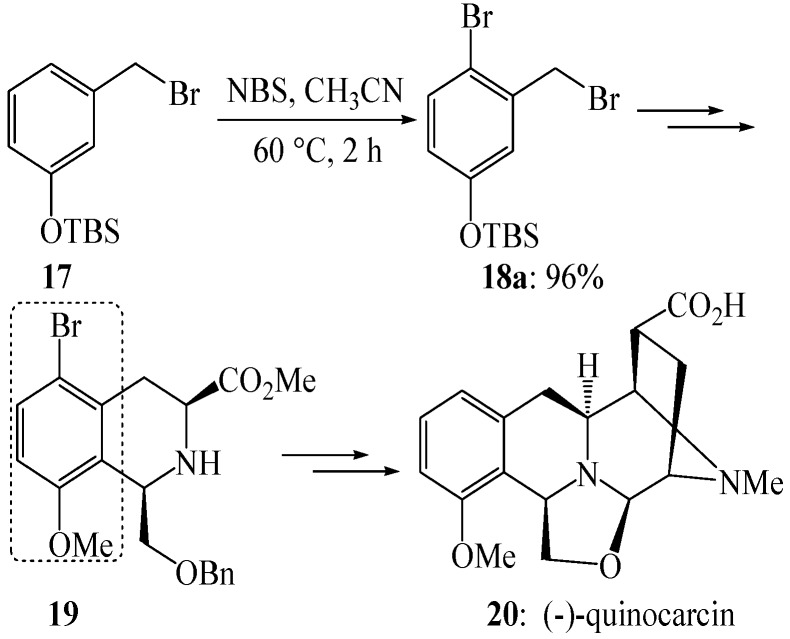
Electrophilic aromatic bromination of compound **17** and its experimental application.

We have an ongoing interest in the development of selective reactions [52,53,54,55,56,57,58,59,60], and have also studied regioselective electrophilic aromatic brominations along with their applications in the total synthesis of antitumor antibiotic tetrahydroisoquinoline alkaloids. For example, transformation of aromatic reactant **17** into **18a**, a key intermediate in one total synthesis of quinocarcin, was accomplished at as low a temperature as possible (Scheme 7) [61]. The electrophilic aromatic bromination at its lowest effective temperature displayed high *para*/*ortho* selectivity and only the *para* isomer was formed. This is because the *tert*-butyldimethylsilyloxyl group is a π-donor substituent that direct the electrophilic aromatic bromination to the positions in the preferential order of *para* > *ortho* > *meta*. The *ortho* isomer was formed when this reaction was performed at a higher temperature. This is because a higher reaction temperature makes more collisions effective, including the collisions at the position *ortho* to the *tert*-butyldimethylsilyloxyl group, and thereby results in a lower positional selectivity. The regiospecific electrophilic aromatic bromination effectively obviated the regioselectivity issue during the following Pictet-Spengler reaction, and thus facilitated an efficient total synthesis of quinocarcin (**20**) [61].

**Scheme 8:**
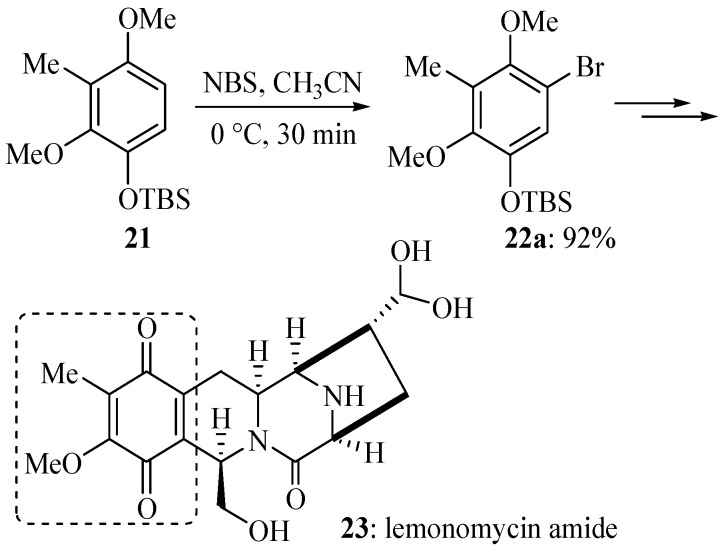
Electrophilic aromatic bromination of compound **21** and its experimental application.

As methoxyl and *tert*-butyldimethylsilyloxyl are quite comparable strong activating substituents, the electrophilic aromatic bromination of compound **21** at its lowest effective temperature was found to be highly *para*-selective with respect to a strong activating substituent to afford exclusively isomer **22a** in 92% yield (Scheme 8) [62], which supports the positional selectivity mentioned previously. It is noteworthy that this electrophilic aromatic bromination only displays high positional selectivity at its lowest effective temperature, and another isomer was also formed when the reaction was performed at a higher temperature such as room temperature. The regiospecific electrophilic aromatic bromination facilitated the total synthesis of the antitumor antibiotic lemonomycin amide (**23**) [62].

**Scheme 9:**
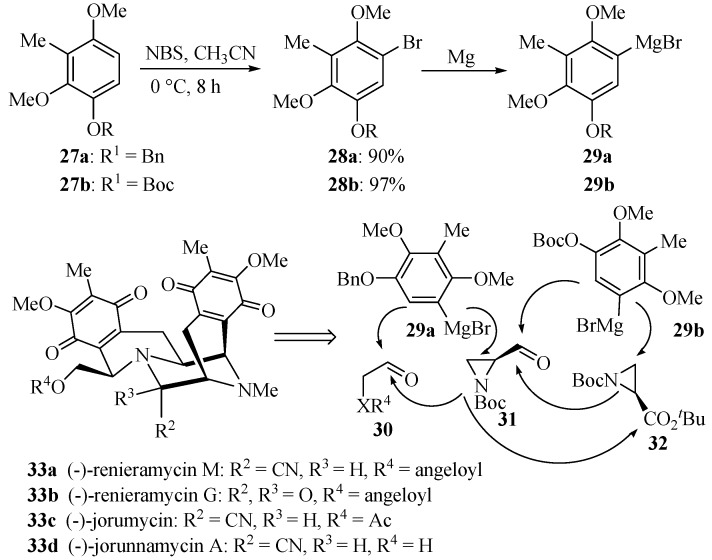
Electrophilic aromatic bromination of compounds **27** and their experimental application.

Regioselective electrophilic aromatic brominations have also facilitated the total syntheses of (−)-renieramycin M (**33a**, Scheme 9), (−)-renieramycin G (**33b**), (−)-jorumycin (**33c**) and (−)-jorunnamycin A (**33d**) [63]. As shown in Scheme 9, treatment of compounds **27a**–**b** with *N*-bromosuccinimide in acetonitrile at their lowest effective temperature for 8 h efficiently afforded aryl bromides **28a**–**b** that were used as precursors to key Grignard reagents **29a**–**b**. By exploring the double nucleophilicity of Grignard reagents **29a**–**b** and triple reactivity of aziridines **31**–**32**, convergent and versatile syntheses of the above four antitumor antibiotic marine natural products were developed [63].

To explain the positional selectivity in an electrophilic aromatic bromination, valence bond theory, resonance theory and frontier molecular orbital theory have been used, and the related electrostatic potentials, charge distribution, electrophile affinity and local ionization energy have been calculated [31,32,33,34,35,36,37]. Moreover, various calculation methods and reaction pathways have also been proposed [31,32,33,34,35,36,37]. Although the accurate positional selectivity in an electrophilic aromatic bromination is unclear at this time, more and more related endeavors would complement each other to lead to a better understanding of the controlling elements behind the corresponding experimental results.

As many factors are usually concurrently involved in an electrophilic aromatic bromination, it seems difficult to evaluate the individual effects separately. However, the electrophilic aromatic bromination could be driven toward the desired product if each individual factor is turned the right way. For example, many π-donor substituents make aromatic reactants nucleophilic and reactive to electrophiles, and thereby make many collisions effective and result in a lower *para*/*ortho* selectivity [29]. One resolution is to perform the electrophilic aromatic bromination at its lowest effective temperature that would decrease the probabilities of effective collisions and increase the *para*/*ortho* selectivity.

## 3. Experimental

### 3.1. General Information

Common reagents and materials were purchased from commercial sources and purified by recrystallization or distillation. Where necessary, organic solvents were routinely dried and/or distilled prior to use and stored over molecular sieves under argon. Organic extracts were, in general, dried over anhydrous sodium sulfate (Na_2_SO_4_). TLC plates were visualized by exposure to ultraviolet light (UV). ^1^H and ^13^C-NMR spectra were measured by Bruker AVANCE III (Bruker; Fallanden, Switzerland) 400 MHz. Chemical shifts for protons are reported in parts per million (δ scale) downfield from tetramethylsilane and are referenced to residual protium in the NMR solvents (CHCl_3_: δ 7.26). Chemical shifts for carbon resonances are reported in parts per million (δ scale) downfield from tetramethylsilane and are referenced to the carbon resonances of the solvent (CDCl_3_: δ 77.0). Data are represented as follows: chemical shift, multiplicity (s = singlet, d = doublet, t = triplet, q = quartet, m = multiplet, br = broad), coupling constant in Hertz (Hz), and integration. IR spectra were recorded on a Nicolet 380 FT-IR spectrometer (Thermo Electron Corp., San Jose, CA, USA).

### 3.2. Calculations

The *ab initio* calculations of the arenium ion in electrophilic aromatic brominations have been performed via the GAUSSIAN 09 program package [47]. The calculations were carried out within the framework of MP2 methodology using 6-311++G (*d*, *p*) basis set. The vibration frequency is calculated for all structures involved to examine the structural stability. Charge densities were calculated from the electrostatic potential by the method of Merz-Kollman [64] and illustrated in Figure 2b. The sign of the charges illustrated in these figures indicates whether the atom is a donor (a cation with positive value) or an acceptor (an anion with negative value).

### 3.3. Electrophilic Aromatic Bromination of Compound **5**

To a solution of compound **5** (99%, 108.9 μL, 1.0 mmol) in acetonitrile (MeCN, 2 mL) at 0 °C was added *N*-bromosuccinimide (NBS, 98%, 181.7 mg, 1.0 mmol) in one portion. The resulting mixture was allowed to come to room temperature, stirred at room temperature overnight, quenched with water (10 mL), and extracted with dichloromethane (CH_2_Cl_2_, 3 × 10 mL). The combined organic layers were washed with brine, dried over anhydrous sodium sulfate (Na_2_SO_4_), filtered, and concentrated. The residue was purified by column chromatography on silica gel (100–200 mesh) to afford exclusively 1-bromo-4-methoxybenzene (**6a**, 179.5 mg) in 96% yield. Pale yellow oil; ^1^H-NMR (400 MHz, CDCl_3_) δ 7.38 (d, *J* = 8.8, 2H), 6.79 (d, *J* = 8.8, 2H), 3.78 (s, 3H); ^13^C-NMR (100 MHz, CDCl_3_) δ 158.7, 132.2, 115.7, 112.8, 55.4; FTIR (film): 3036, 2937, 1490, 1075, 1012, 800 cm^−1^. Anal. Calcd. for C_7_H_7_BrO_3_: C, 44.95; H, 3.77. Found: C, 44.88; H, 3.93.

### 3.4. Electrophilic Aromatic Bromination of Compound **17**

To a solution of compound **17** (301.3 mg, 1.0 mmol) in acetonitrile (MeCN, 2 mL) at room temperature was added *N*-bromosuccinimide (NBS, 98%, 181.7 mg, 1.0 mmol) in one portion. The resulting mixture was stirred at 60 °C for 2 h, quenched with water (10 mL), and extracted with dichloromethane (CH_2_Cl_2_, 3 × 10 mL). The combined organic layers were washed with brine, dried over anhydrous sodium sulfate (Na_2_SO_4_), filtered, and concentrated. The residue was purified by column chromatography on silica gel (100–200 mesh) to afford exclusively 6-bromo-3-*tert*-butyldimethylsilyloxybenzyl bromide (**18a**, 364.9 mg) in 96% yield. Pale yellow oil; ^1^H-NMR (300 MHz, CDCl_3_) δ 7.40 (d, 1H, *J* = 8.7 Hz), 6.95 (d, 1H, *J* = 1.8 Hz), 6.66 (dd, 1H, *J* = 8.7, 1.8 Hz), 4.53 (s, 2H), 0.99 (s, 9H), 0.21 (s, 6H); ^13^C-NMR (75 MHz, CDCl_3_) δ 155.3, 137.8, 133.9, 122.9, 122.0, 115.5, 33.3, 25.6, 18.2, −4.4; FTIR (film) 2928, 2856, 1470, 1290, 1246, 1171, 981, 834, 816, 779, 668 cm^−^^1^. Anal. calcd. for C_13_H_20_Br_2_OSi: C, 41.07; H, 5.30. Found: C, 40.99; H, 5.36.

### 3.5. Electrophilic Aromatic Bromination of Compound **21**

To a solution of compound **21** (282.5 mg, 1.0 mmol) in acetonitrile (MeCN, 2 mL) at −10 °C was added *N*-bromosuccinimide (NBS, 98%, 181.7 mg, 1.0 mmol) in one portion. The resulting mixture was stirred at 0 °C for 0.5 h, quenched with water (10 mL), and extracted with dichloromethane (CH_2_Cl_2_, 3 × 10 mL). The combined organic layers were washed with brine, dried over anhydrous sodium sulfate (Na_2_SO_4_), filtered, and concentrated. The residue was purified by column chromatography on silica gel (100–200 mesh) to afford exclusively compound **22a** (332.4 mg) in 92% yield. Pale yellow oil; ^1^H-NMR (300 MHz, CDCl_3_) δ 6.89 (s, 1H), 3.75 (s, 3H), 3.73 (s, 3H), 2.23 (s, 3H), 1.00 (s, 9H), 0.19 (s, 6H); ^13^C-NMR (75 MHz, CDCl_3_) δ 149.9, 149.5, 145.7, 127.0, 121.8, 110.6, 60.3, 59.8, 25.6, 18.1, 10.1, −4.7; FTIR (film): 2954, 2930, 2895, 2858, 1470, 1417, 1401, 1315, 1252, 1234, 1205, 1171, 1097, 1049, 1001, 860, 837, 800, 781, 683 cm^−1^. Anal. calcd. for C_15_H_25_BrO_3_Si: C, 49.86; H, 6.97. Found: C, 49.89; H, 6.87.

### 3.6. Electrophilic Aromatic Bromination of Compound **27*a***

To a solution of compound **27a** (258.4 mg, 1.0 mmol) in acetonitrile (MeCN, 2 mL) at −10 °C was added *N*-bromosuccinimide (NBS, 98%, 181.7 mg, 1.0 mmol) in one portion. The resulting mixture was stirred at 0 °C for 8 h, quenched with water (10 mL), and extracted with dichloromethane (CH_2_Cl_2_, 3 × 10 mL). The combined organic layers were washed with brine, dried over anhydrous sodium sulfate (Na_2_SO_4_), filtered, and concentrated. The residue was purified by column chromatography on silica gel (100–200 mesh) to afford compound **28a** (303.5 mg) in 90% yield. White solids; mp = 34–36 °C; ^1^H-NMR (300 MHz, CDCl_3_) δ 7.47–7.13 (m, 5H), 6.90 (s, 1H), 4.92 (s, 2H), 3.72 (s, 3H), 3.64 (s, 3H), 2.15 (s, 3H); ^13^C-NMR (75 MHz, CDCl_3_) δ 149.7, 148.8, 147.8, 136.6, 128.5, 128.4, 127.9, 127.3, 115.5, 100.5, 71.2, 60.31, 60.25, 10.1; FTIR (film): 2933, 2866, 1476, 1452, 1421, 1402, 1379, 1313, 1260, 1232, 1175, 1087, 1068, 1028, 1000, 968, 908, 888, 826, 790, 776, 732, 695 cm^−1^. HRMS (TOF MS ES^+^) *m*/*z*: Calcd for C_16_H_17_O_3_NaBr [M + Na]^+^: 359.0259 & 361.0238. Found: 359.0263 and 361.0246.

### 3.7. Electrophilic Aromatic Bromination of Compound **27*b***

To a solution of compound **27b** (268.4 mg, 1.0 mmol) in acetonitrile (MeCN, 2 mL) at −10 °C was added *N*-bromosuccinimide (NBS, 98%, 181.7 mg, 1.0 mmol) in one portion. The resulting mixture was stirred at 0 °C for 8 h, quenched with water (10 mL), and extracted with dichloromethane (CH_2_Cl_2_, 3 × 10 mL). The combined organic layers were washed with brine, dried over anhydrous sodium sulfate (Na_2_SO_4_), filtered, and concentrated. The residue was purified by column chromatography on silica gel (100–200 mesh) to afford compound **28b** (336.7 mg) in 97% yield. Yellow oil; ^1^H-NMR (300 MHz, CDCl_3_) δ 7.21 (s, 1H), 3.79 (s, 3H), 3.78 (s, 3H), 2.26 (s, 3H), 1.55 (s, 9H); ^13^C-NMR (75 MHz, CDCl_3_) δ 153.9, 151.4, 150.3, 140.7, 127.6, 124.0, 110.9; FTIR (film): 2980, 2937, 1760, 1471, 1417, 1370, 1272, 1255, 1223, 1147, 1099, 1067, 1004, 969, 882, 753 cm^−1^. HRMS (TOF MS ES^+^) *m*/*z*: Calcd. for C_14_H_19_BrO_5_Na [M + Na]^+^: 369.0314. Found: 369.0318.

## 4. Conclusions

In summary, the results based on *ab initio* calculations provide clear indication for the positional selectivity tendencies in an electrophilic aromatic bromination. Without considering any other factors, a π-donor substituent would direct an electrophilic aromatic bromination to the position in the preferential order of *para* > *ortho* > *meta*, whereas a π-acceptor substituent would direct this substitution to the position in the preferential order of *meta* > *ortho* > *para*. Although direct comparison is not always valid due to the different reaction conditions, the tendency is nevertheless quite clear. The regioselective electrophilic aromatic bromination has facilitated the synthesis of various bioactive natural products, which might allow organic chemists to think more clearly about their related research. Moreover, the theoretical analysis could also be used for understanding the regioselectivity of electrophilic aromatic brominations in the related publications.

## Supplementary Materials

Supplementary materials can be accessed at: http://www.mdpi.com/1420-3049/19/3/3401/s1.
