# Supplementary material for: Regioselective Electrophilic Aromatic Bromination: Theoretical Analysis and Experimental Verification
Source: Molecules. 2014 Mar 20;19(3):3401–16. doi: 10.3390/molecules19033401 (PMC6271510; doi:10.3390/molecules19033401)

Supplementary Materials

Table of Contents

1. NMR spectra	**S1**
2. Diagram of Calculation Targets	**S2**

1. NMR Spectra

Compound **6a** ^{1}H-NMR (400 MHz, $CDCl_3$).

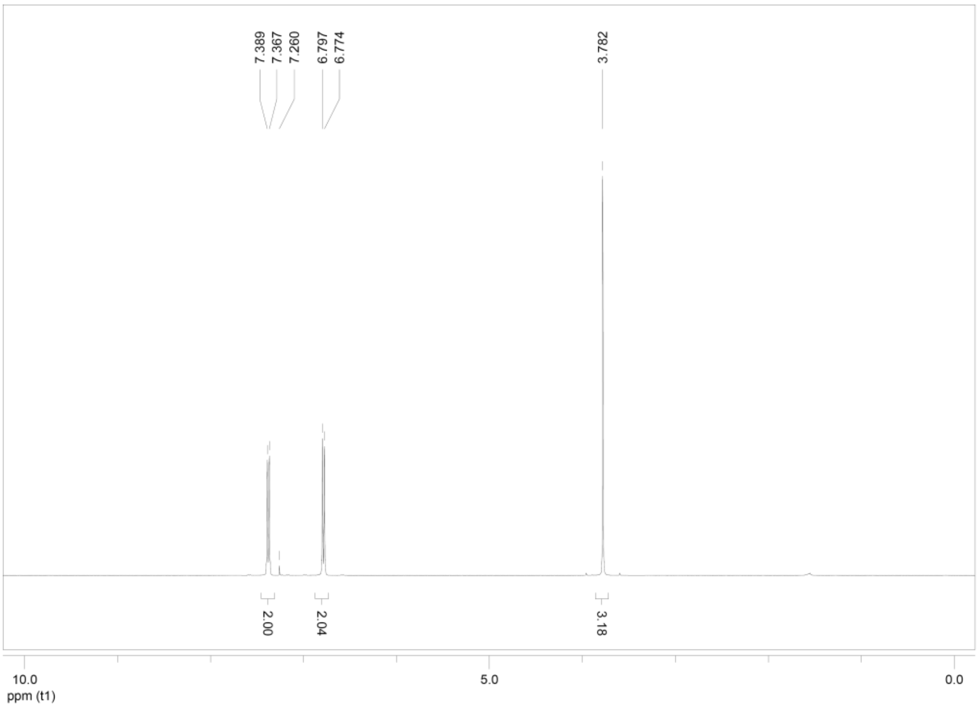

Compound **6a** ^{13}C-NMR (100 MHz, $CDCl_3$).

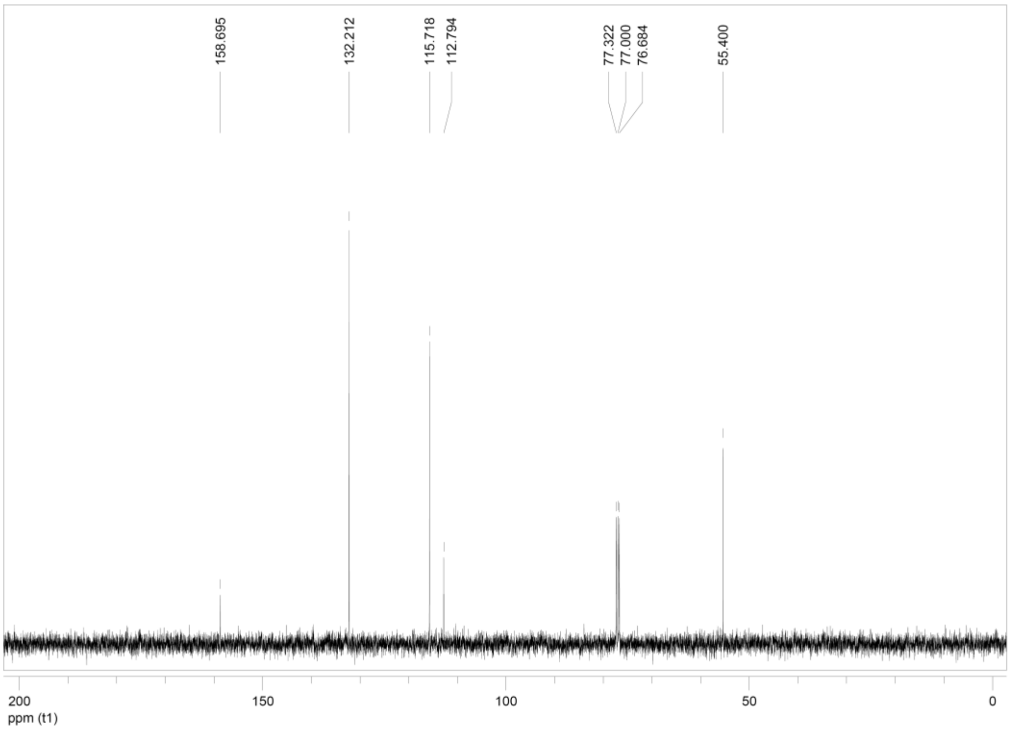

For the NMR Spectra of compound **18a**, see the Supporting Information of the following paper:

1. Wu, Y.C.; Liron, M.; Zhu, J.P. Asymmetric total synthesis of (−)-quinocarcin. *J. Am. Chem. Soc.* **2008**, *130*, 7148–7152.

For the NMR Spectra of compound **22a**, see the Supporting Information of the following paper:

2. Wu, Y.C.; Bernadat, G.; Masson, G.; Couturier, C.; Schlama, T.; Zhu, J.P. Synthetic studies on (−)-lemonomycin: An efficient asymmetric synthesis of lemonomycinone amide. *J. Org. Chem.* **2009**, *74*, 2046–2052.

For the NMR Spectra of compound **28a** and compound **28b**, see the Supporting Information of the following paper:

3. Wu Y.C.; Zhu, J.P. Asymmetric total syntheses of (−)-renieramycin M and G and (−)-jorumycin using aziridine as a lynchpin. *Org. Lett.* **2009**, *11*, 5558–5561.

2. Diagram of Calculation Targets

Figure S1. Charge distribution of the arenium ion.

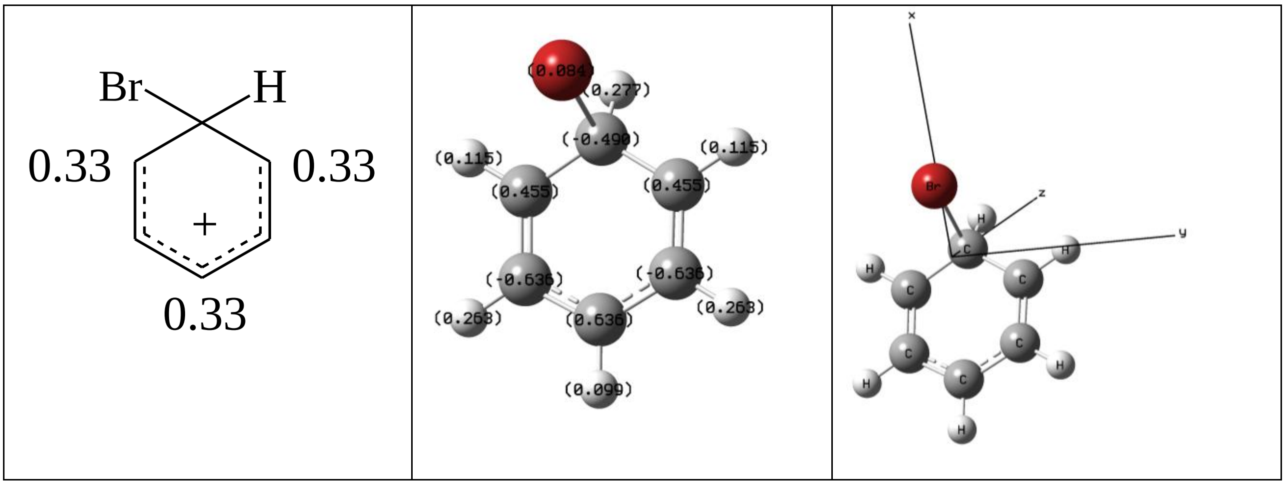

Figure S2. Total of electronic energy (Hartree) of the transition-state of the *ortho*-isomer product in the electrophilic aromatic bromination of anisole.

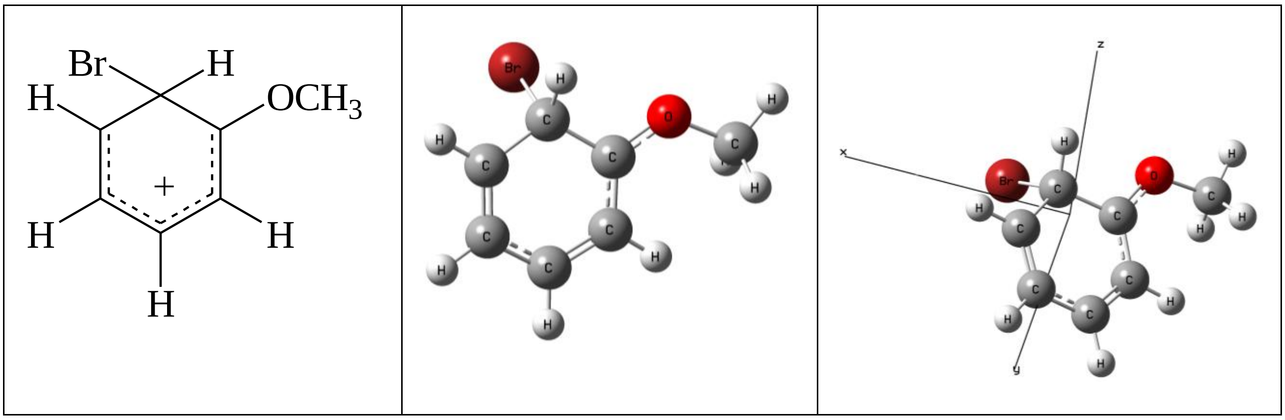

Figure S3. Total of electronic energy (Hartree) of the transition-state of the *meta*-isomer product in the electrophilic aromatic bromination of anisole.

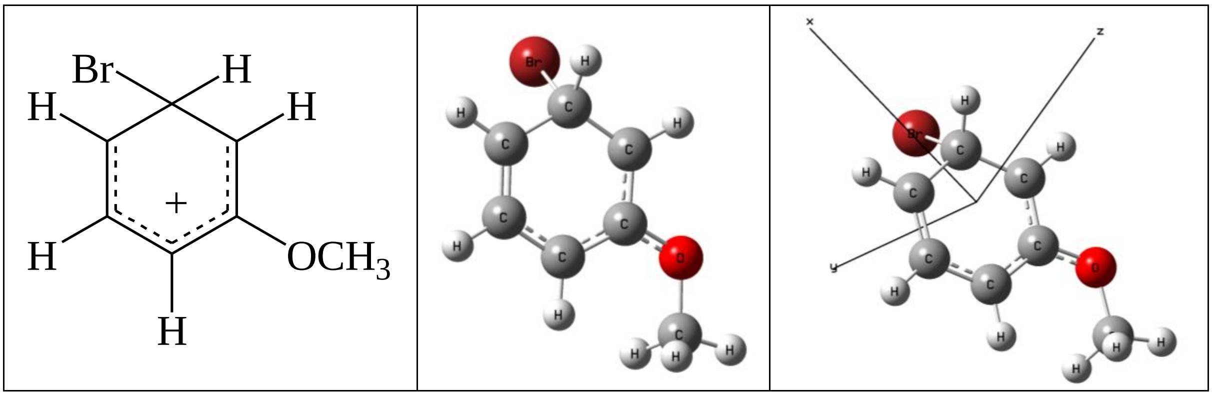

Figure S4. Total of electronic energy (Hartree) of the transition-state of the *para*-isomer product in the electrophilic aromatic bromination of anisole.

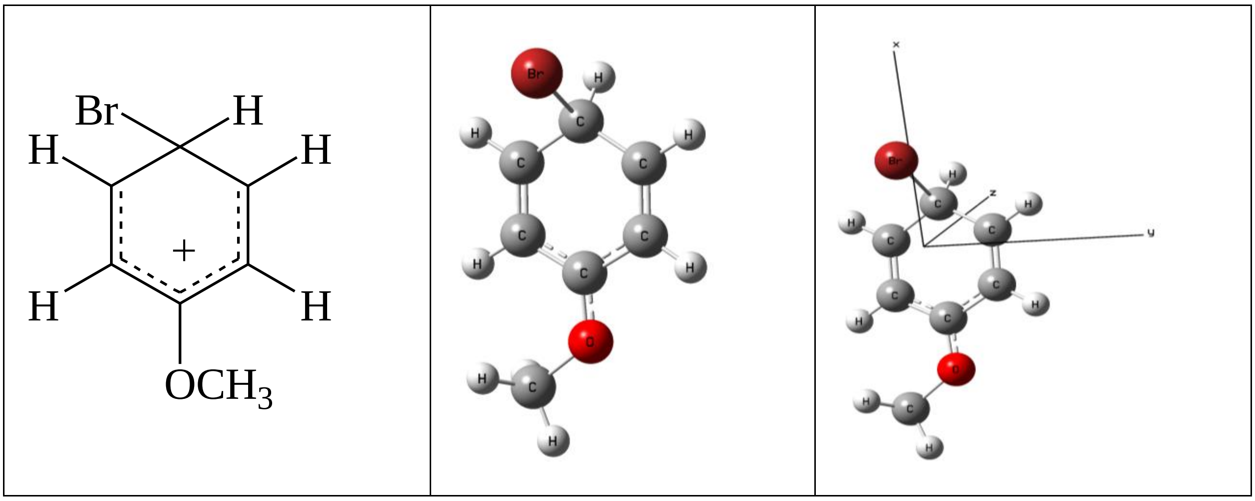

Figure S5. Total of electronic energy (Hartree) of the *ortho*-isomer product in the electrophilic aromatic bromination of anisole.

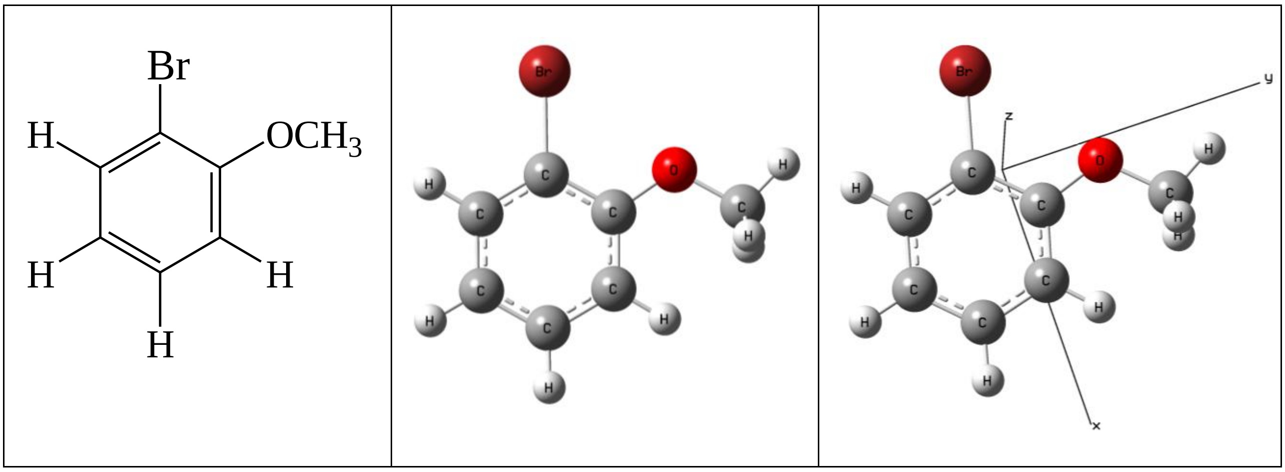

Figure S6. Total of electronic energy (Hartree) of the *meta*-isomer product in the electrophilic aromatic bromination of anisole.

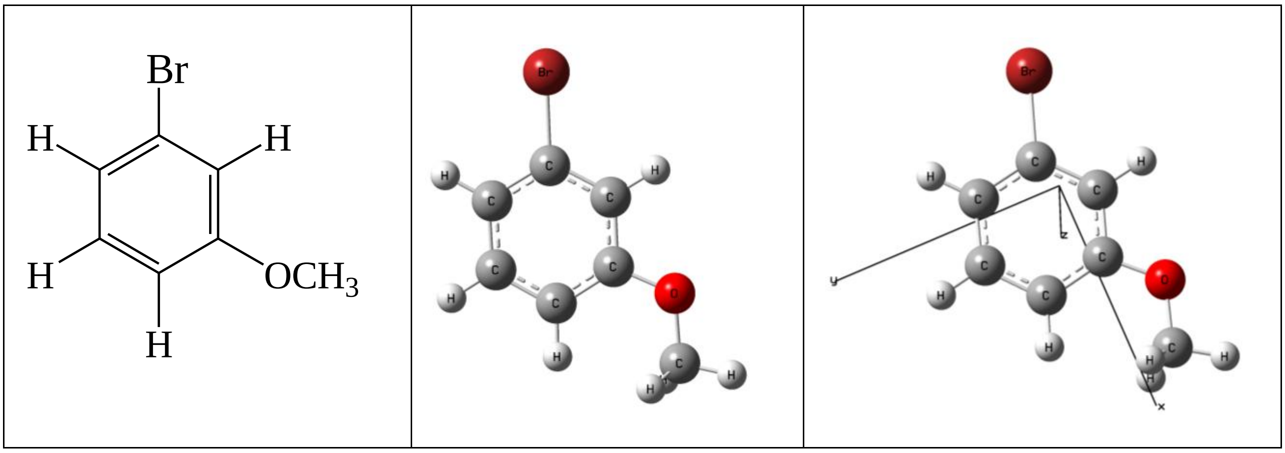

Figure S7. Total of electronic energy (Hartree) of the *para*-isomer product in the electrophilic aromatic bromination of anisole.

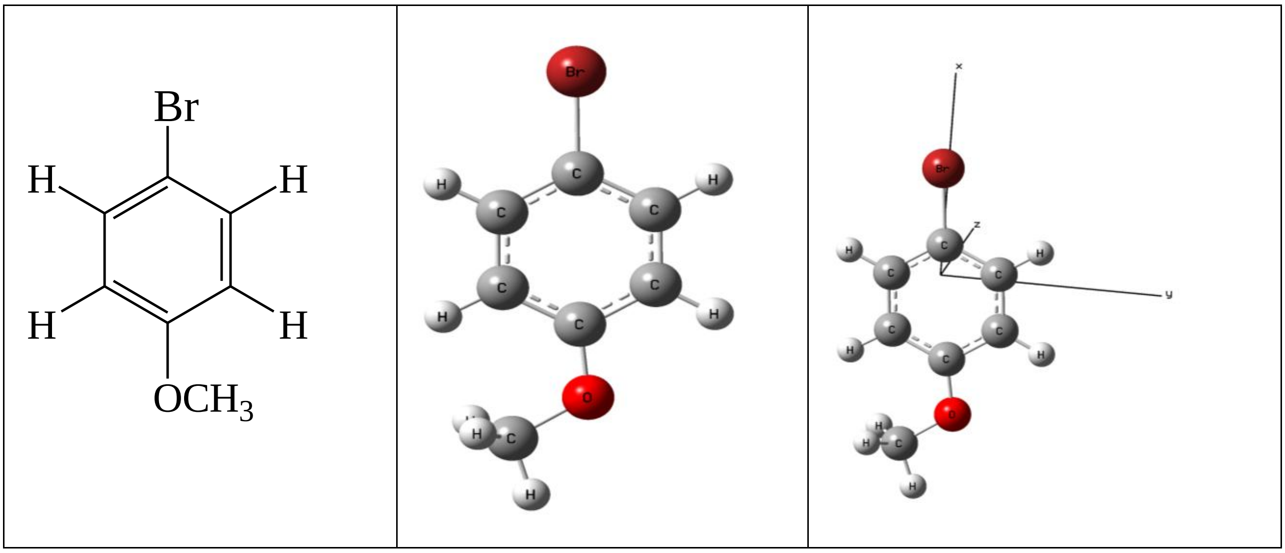

Supplement: Supplementary file 1 [file molecules-19-03401-s001.pdf]
